# Cellular phosphatidic acid sensor, α-synuclein N-terminal domain, detects endogenous phosphatidic acid in macrophagic phagosomes and neuronal growth cones

**DOI:** 10.1016/j.bbrep.2020.100769

**Published:** 2020-05-20

**Authors:** Haruka Yamada, Fumi Hoshino, Qiang Lu, Fumio Sakane

**Affiliations:** Department of Chemistry, Graduate School of Science, Chiba University, Chiba, 263-8522, Japan

**Keywords:** Phosphatidic acid, Lipid sensor, α-Synuclein, Phagosome, Growth cone, Phospholipase D, Diacylglycerol kinase, α-Syn, α-synuclein, α-Syn-N, N-terminal region of α-Syn, DGK, diacylglycerol kinase, DMEM, Dulbecco's modified Eagle's medium, F-actin, filamentous actin, FIPI, 5-fluoro-2-indolyl deschlorohalopemide, LPA, lysophosphatidic acid, LPAAT, LPA acyltransferase, Myr, myristoylated, PA, phosphatidic acid, PC, phosphatidylcholine, PABD, phosphatidic acid-binding domain, PLD, phospholipase D

## Abstract

Phosphatidic acid (PA) is the simplest phospholipid and is involved in the regulation of various cellular events. Recently, we developed a new PA sensor, the N-terminal region of α-synuclein (α-Syn-N). However, whether α-Syn-N can sense physiologically produced, endogenous PA remains unclear. We first established an inactive PA sensor (α-Syn-N-KQ) as a negative control by replacing all eleven lysine residues with glutamine residues. Using confocal microscopy, we next verified that α-Syn-N, but not α-Syn-N-KQ, detected PA in macrophagic phagosomes in which PA is known to be enriched, further indicating that α-Syn-N can be used as a reliable PA sensor in cells. Finally, because PA generated during neuronal differentiation is critical for neurite outgrowth, we investigated the subcellular distribution of PA using α-Syn-N. We found that α-Syn-N, but not α-Syn-N-KQ, accumulated at the peripheral regions (close to the plasma membrane) of neuronal growth cones. Experiments using a phospholipase D (PLD) inhibitor strongly suggested that PA in the peripheral regions of the growth cone was primarily produced by PLD. Our findings provide a reliable sensor of endogenous PA and novel insights into the distribution of PA during neuronal differentiation.

## 1 Introduction

Phosphatidic acid (PA) is a phospholipid that regulates many cellular events, including phagocytosis [1], adhesion/migration [2], proliferation [3], and differentiation [4], as a lipid second messenger. PA is produced by multiple pathways, such as the phosphorylation of diacylglycerol by diacylglycerol kinase (DGK) [[5], [6], [7], [8]], the hydrolysis of phosphatidylcholine (PC) by phospholipase D (PLD) [9], and the transacylation of lysoPA (LPA) by LPA acyltransferase (LPAAT) [10]. DGK [11,12], PLD [9,13,14] and LPAAT [15,16] are involved in the pathogenesis of a wide variety of diseases, such as cancer, epilepsy, obsessive-compulsive disorder, bipolar disorder, neurodegenerative disorders (Parkinson's and Alzheimer's diseases), autoimmunity, cardiac hypertrophy, hypertension and type II diabetes. Consequently, sensing PA produced by these enzymes in cells is important for understanding diverse biological and pathological phenomena. However, most PA-binding domains (PABDs) reported to date exhibit their own subcellular localization to membranes, including the Golgi apparatus and plasma membrane, in a cell stimulation (PA generation)-independent manner [17,18]. The cell stimulation-independent localization to membranes diminishes their functions as PA sensors. Because a reliable and widely applicable PA sensor has not been established to date, developing an excellent PA sensor is an urgent issue.

Recently, we determined that α-synuclein (α-Syn) selectively and intensely interacted with PA *in vitro* [19] and that the N-terminal region of α-Syn (α-Syn-N) is a PABD [20]. Notably, α-Syn-N did not show significant membrane localization in quiescent cells [20]. In addition, α-Syn-N colocalized with various overexpressed PA-generating enzymes, such as DGKβ, phorbol ester-stimulated DGKγ, myristoylated (Myr)-DGKζ and PLD2, but not with a phosphatidylinositol 4,5-bisphosphate-producing enzyme, phosphatidylinositol 4-phosphate 5-kinase, in an activity-dependent manner [20]. These results indicate that α-Syn-N can bind to intracellular PA, but not the PA-producing enzyme proteins themselves. Therefore, α-Syn-N can be used as a reliable and widely applicable PA sensor in cells. However, whether α-Syn-N can sense physiologically produced, endogenous PA remains unclear.

In the present study, we first established an inactive PA sensor (α-Syn-N-KQ) as a negative control by replacing all (eleven) lysine residues with glutamine residues. We next confirmed that α-Syn-N, but not α-Syn-N-KQ, recognized PA in macrophagic phagosomes where PA is known to be enriched [1,17], further indicating that α-Syn-N can be used as a reliable PA sensor for endogenous PA in cells.

PA produced by PLD and DGK is linked to neurite outgrowth [[21], [22], [23], [24], [25]]. However, where PA exists in neurites is not clear. Therefore, we investigated the subcellular localization of PA during neuroblastoma cell differentiation using α-Syn-N.

## 2 Materials and methods

### 2.1 Materials

The mouse anti-6 × His-tag (D291-3) antibody was purchased from Medical and Biological Laboratories (Nagoya, Japan). A peroxidase-conjugated goat anti-mouse IgG antibody was purchased from Jackson ImmunoResearch Laboratories (West Grove, PA, USA). A mouse anti-FLAG M2 antibody was obtained from Sigma-Aldrich (St. Louis, MO, USA). The Alexa Fluor 594 goat anti-mouse IgG (A11005) and Alexa Fluor 594 phalloidin (A12381) were purchased from Thermo Fisher Scientific (Waltham, MA, USA). 1-Palmitoyl-2-oleoyl-sn-glycero-3-phosphocholine (16:0/18:1-PC) was purchased from Sigma-Aldrich. 1,2-dioleoyl-sn-glycero-3-phosphate (18:1/18:1-PA) was purchased from Avanti Polar Lipids (Alabaster, AL, USA). Cholesterol was obtained from Wako Pure Chemical Industries (Osaka, Japan).

### 2.2 Plasmids constructs

p6 × His-SUMO-α-Syn-N and pAcGFP-α-Syn-N were generated previously [20]. To construct p6 × His-SUMO-α-Syn-N-KQ and pAcGFP-α-Syn-N-KQ, cDNA in which all lysine codons (AAA and AAG) were converted to a glutamine codon (CAG) was synthesized by Eurofins Genomics K.K. (Tokyo, Japan); the modified cDNA was then inserted into the p6 × His-SUMO vector at the *Nde*I/*Xho*I sites, and the pAcGFP-C1 vector (Takara-Clontech (Kusatsu, Japan)) at the *Eco*RI/*Sal*I sites. p3 × FLAG-Myr-DGKζ was constructed by inserting a PCR fragment encoding the full-length human DGKζ isoform 1 into the N-myristoylation sequence-fused p3 × FLAG-CMV-7.1 vector (Sigma-Aldrich) at the *Eco*RI/*Sal*I sites. The N-myristoylation sequence was inserted into the translation initiation site of 3 × FLAG in the p3 × FLAG-CMV-7.1 [26]. p3 × FLAG-DGKζ was constructed by inserting the PCR fragment encoding full-length mouse DGKζ isoform 1 into p3 × FLAG-CMV-7.1 vector at the *Eco*RI/*Sal*I sites.

### 2.3 Protein expression and purification

The expression and purification (Ni^2+^-NTA affinity chromatography) of 6 × His-SUMO-tagged proteins were performed as described previously [20]. For the liposome co-sedimentation assay, the purified proteins were dialyzed in HEPES buffer (25 mM HEPES, pH 7.4, 100 mM NaCl). The protein concentration was measured with a bicinchoninic acid protein assay kit (Thermo Fisher Scientific).

### 2.4 Liposome co-sedimentation assay

The liposome co-sedimentation assay was performed as described previously [20]. The purified proteins (2.5 μM) were incubated with a PA-containing liposome (cholesterol (50 mol%), 16:0/18:1-PC (35 mol%), 18:1/18:1-PA (15 mol% (150 μM))) and ultracentrifuged. The supernatant and pellet were analyzed by SDS-PAGE, followed by Western blotting.

### 2.5 COS-7 cell culture and transfection

COS-7 cells were maintained and transfected as described previously [20].

### 2.6 RAW264 cell culture and phagocytosis assay

RAW264 macrophage cells were maintained in Dulbecco's modified Eagle's medium (DMEM) (Wako Pure Chemical Industries) supplemented with 10% fetal bovine serum (Biological Industries, Beit-Haemek, Israel), 100 units/ml penicillin, and 100 μg/ml streptomycin (Wako Pure Chemical Industries) at 37 °C in an atmosphere containing 5% CO_2_. The cells were transfected using Lipofectamine 3000 (Thermo Fisher Scientific) as described by the manufacturer. Opsonized beads used for phagocytosis induction were prepared by incubating 3.0 μm polystyrene latex beads (Sigma-Aldrich) and 2 mg/ml normal rabbit IgG (Wako Pure Chemical Industries) at room temperature, and then incubating with goat anti-rabbit IgG-Alexa Fluor 594 (A11012, Thermo Fisher Scientific). The prepared opsonized beads were added to the dish containing RAW264 cells cultured for 24 h after transfection; the cells were incubated for 10 min at 37 °C and fixed by 4% paraformaldehyde.

### 2.7 Neuro-2a cell culture and induction of neurite outgrowth

Neuro-2a neuroblastoma cells were maintained under the same conditions as RAW264 cells. The cells were transfected using Polyfect reagent (Qiagen, Venlo, the Netherlands) as described by the manufacturer. To induce neuronal differentiation, Neuro-2a cells were cultured in serum-free DMEM for 48 h and then fixed by 4% paraformaldehyde.

### 2.8 Confocal laser scanning microscopy

The cells were processed as described previously [27] and were observed using an Olympus FV1000-D (IX81) confocal laser scanning microscope (Olympus, Tokyo, Japan). Images were acquired using FV-10 ASW software (Olympus). The analysis of protein accumulation was performed using ImageJ software (National Institutes of Health, Bethesda, MD, USA) as previously described [20].

### 2.9 Statistical analysis

Data are represented as the means ± SD or ± SEM and were analyzed by Student's t-test, Kolmogorov–Smirnov test or Kruskal-Wallis followed by Dunn's multiple comparison test for multiple comparisons using GraphPad Prism 8 (GraphPad software, San Diego, CA, USA) to determine any significant differences. *P* < 0.05 was considered significant.

## 3 Results

### 3.1 α-Syn-N-KQ mutant does not bind to PA

**Fig. 1:**
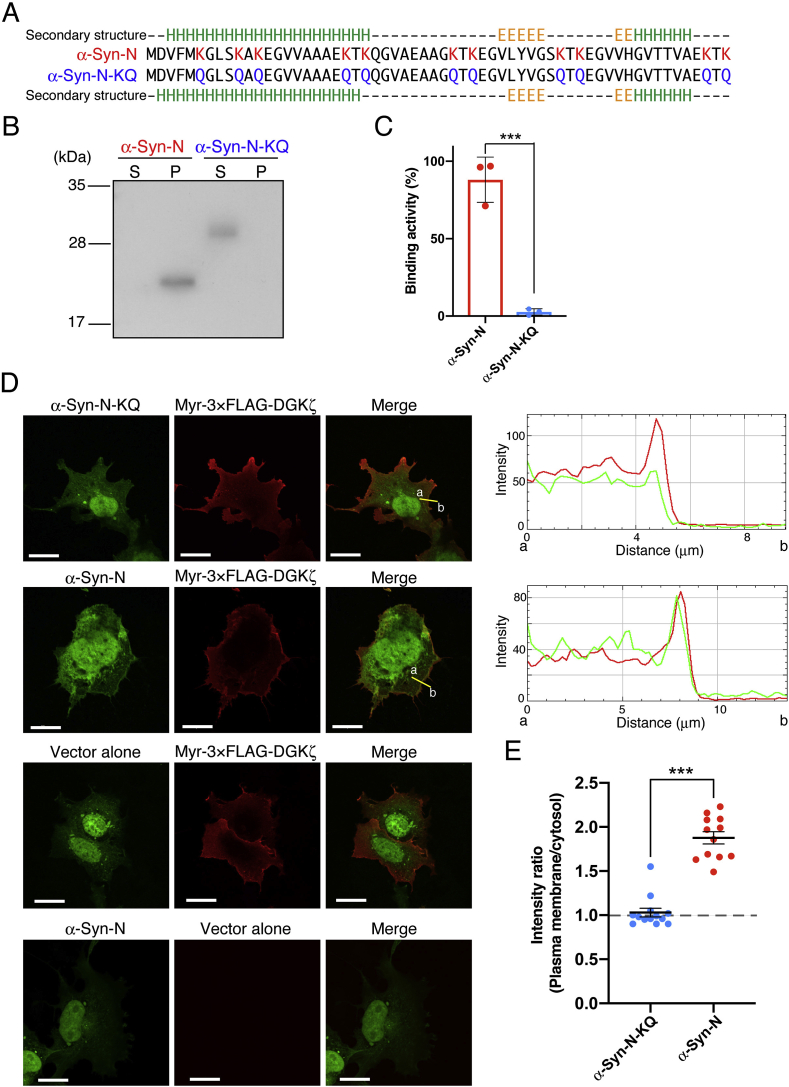
PA binding activity of the α-Syn-N-KQ mutant. (A) Amino acid sequences and secondary structure predictions of α-Syn-N and α-Syn-N-KQ. Secondary structure predictions were calculated by Jpred 4 software (http://www.compbio.dundee.ac.uk/jpred/). H, α-helix; E, extended sheet (β-sheet); –, random coil (unstructured region). (B) The purified 6 × His-SUMO-α-Syn-N and KQ proteins were incubated with PA liposome and then separated by ultracentrifugation. SDS-PAGE (15%) was performed, and proteins were detected by anti-6 × His-tag antibody. *S*, supernatant; *P*, precipitate. (C) Binding activity was calculated as the percentage of the precipitate band intensity compared to the total band intensities. Values are presented as the mean ± SD of three independent experiments. (D) Either pAcGFP vector alone, pAcGFP-α-Syn-N or pAcGFP-α-Syn-N-KQ and either p3 × FLAG vector alone or pMyr-3 × FLAG-DGKζ were co-transfected into COS-7 cells. After 24 h, cells were stained with a mouse anti-FLAG monoclonal antibody and a goat anti-mouse IgG-Alexa Fluor 594. The localization of Myr-3 × FLAG-DGKζ, AcGFP-α-Syn-N and AcGFP-α-Syn-N-KQ was quantified using ImageJ software. Representative data from three independent experiments are shown. Scale bars, 20 μm. (E) Quantitative image analysis of α-Syn-N (n = 12) and α-Syn-N-KQ (n = 13) accumulation at the plasma membrane. Each dot shows the plasma membrane:cytosol intensity ratio. Bars, mean ± SEM. ***P < 0.001, Kolmogorov–Smirnov test.

We first generated a negative control of α-Syn-N (α-Syn-N-KQ) by replacing all eleven lysine residues, which are predicted to be critical for PA binding [28], with glutamine residues (Fig. 1A). The predicted secondary structure of α-Syn-N-KQ (Jpred 4 software (http://www.compbio.dundee.ac.uk/jpred/)) was nearly the same as that of α-Syn-N (Fig. 1A). Next, 6 × His-SUMO-α-Syn-N-KQ and 6 × His-SUMO-α-Syn-N were expressed in *E. coli* and then purified by Ni^2+^-affinity chromatography. The molecular mass of 6 × His-SUMO-α-Syn-N-KQ (~29 kDa) was greater than that of 6 × His-SUMO-α-Syn-N (~22 kDa) (Fig. 1B). This discrepancy is probably due to the loss of positive charges. A liposome sedimentation assay of these proteins demonstrated that the PA-binding activity of 6 × His-SUMO-α-Syn-N-KQ was negative (only 2.6 ± 1.7% (mean ± SD) sedimentation) whereas 6 × His-SUMO-α-Syn-N was almost completely sedimented (88.1% ± 11.9% (mean ± SD) sedimentation) (Fig. 1B and C).

In addition to the *in vitro* liposome sedimentation assay, AcGFP-α-Syn-N co-localized with Myr-3 × FLAG-DGKζ at the plasma membrane (plasma membrane/cytosol intensity ratio: 1.88 ± 0.07 (mean ± SEM)) in COS-7 cells as previously reported [20], whereas AcGFP-α-Syn-N-KQ did not (plasma membrane/cytosol intensity ratio: 1.03 ± 0.05 (mean ± SEM)) (Fig. 1D and E). These results indicate that α-Syn-N-KQ can be used as a negative control of the α-Syn-N PA sensor *in vitro* and in cells.

### 3.2 α-Syn-N detects PA in macrophagic phagosomes

**Fig. 2:**
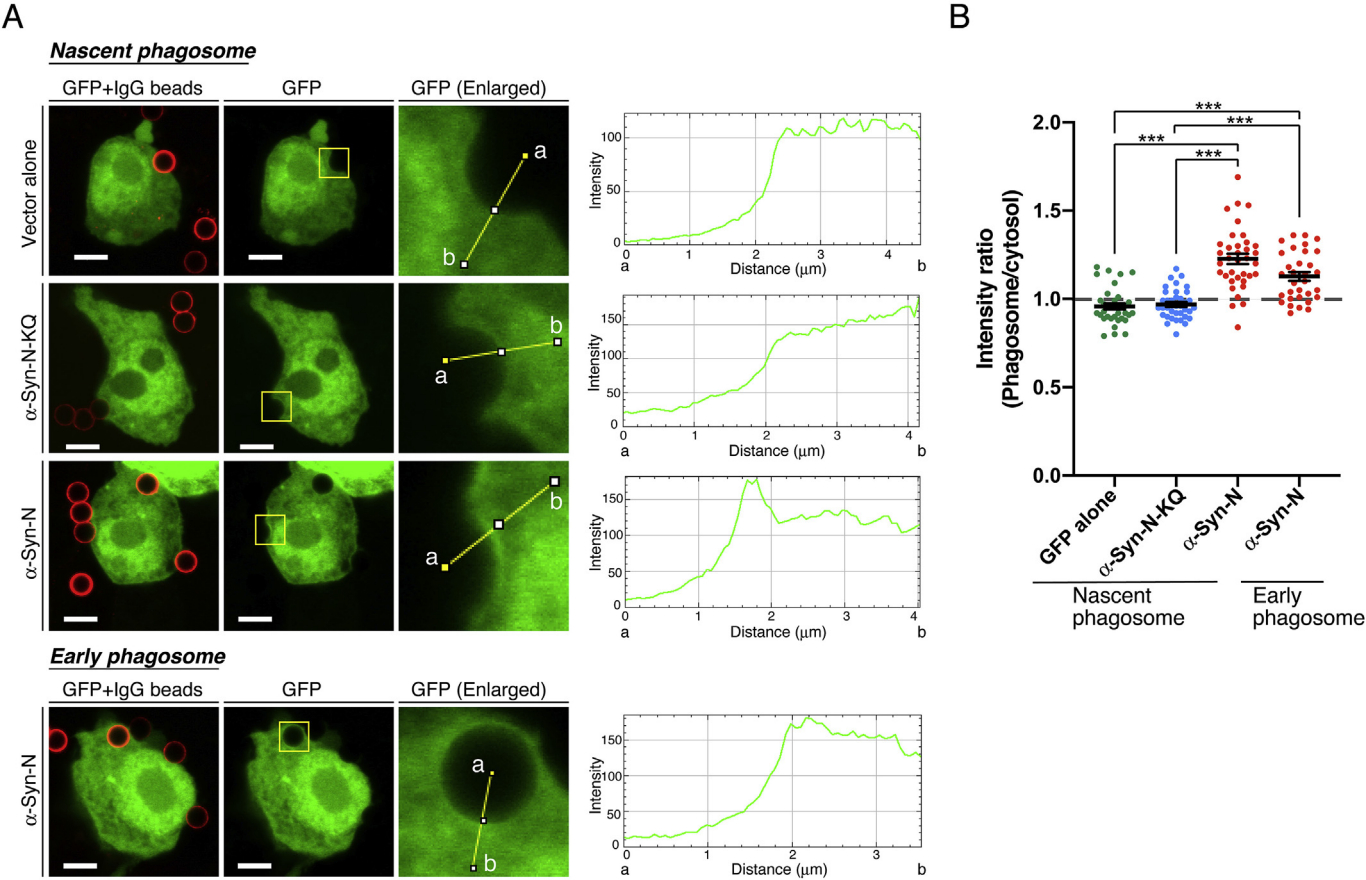
Localization of α-Syn-N in macrophages during phagosome formation. (A) AcGFP alone, AcGFP-α-Syn-N or AcGFP-α-Syn-N-KQ-expressing RAW264 cells were treated with IgG-opsonized beads for 10 min. Protein localization around phagosome was quantified by ImageJ software. Representative data from three independent experiments are shown. Scale bars, 5 μm. (B) Quantitative image analysis of AcGFP alone (n = 35), AcGFP-α-Syn-N-KQ (n = 37), AcGFP-α-Syn-N (Nascent phagosomes) (n = 36) and AcGFP-α-Syn-N (Early phagosomes) (n = 32) accumulation at the phagosome. Each dot shows the phagosome:cytosol intensity ratio. Bars, mean ± SEM. ***P < 0.001, Kruskal-Wallis followed by Dunn's multiple comparison tests.

We next examined whether α-Syn-N recognizes PA in macrophagic phagosomes, in which PA is well known to be enriched [1,17]. As shown in Fig. 2, AcGFP-α-Syn-N intensely recognized the membranes of nascent phagosomes in RAW264 macrophage cells (phagosome/cytosol intensity ratio: 1.23 ± 0.03 (mean ± SEM). However, AcGFP alone (phagosome/cytosol intensity ratio: 0.96 ± 0.01 (mean ± SEM)) and AcGFP-α-Syn-N-KQ (phagosome/cytosol intensity ratio: 0.97 ± 0.01 (mean ± SEM)) failed to be located at nascent phagosome membranes. AcGFP-α-Syn-N was less intensely detected in early (internalized) endosome membranes (phagosome/cytosol intensity ratio: 1.13 ± 0.02 (mean ± SEM). This localization pattern of AcGFP-α-Syn-N is identical to the PA distribution in nascent and early phagosome membranes during phagocytosis as previously reported [1]. Therefore, these results indicate that AcGFP-α-Syn-N is a reliable sensor for endogenous PA and that AcGFP-α-Syn-N-KQ is a negative control of AcGFP-α-Syn-N.

### 3.3 α-Syn-N detects PA in neuronal growth cones

**Fig. 3:**
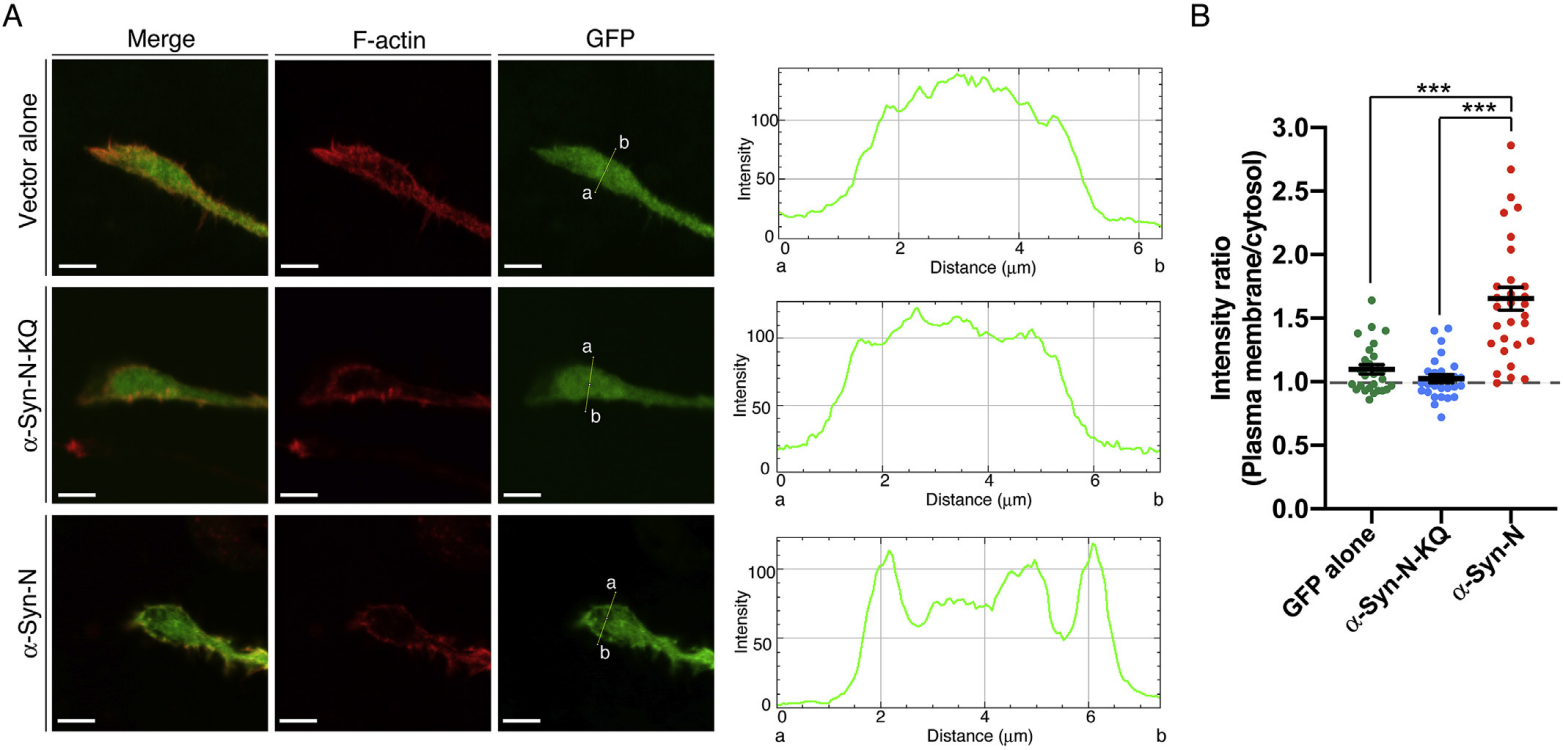
Localization of α-Syn-N in neuronal growth cones. (A) Localization of AcGFP alone, AcGFP-α-Syn-N or AcGFP-α-Syn-N-KQ in the growth cone of Neuro-2a cells. Cells were stained with phalloidin, which recognizes F-actin. Representative data from three independent experiments are shown. Scale bars, 5 μm. (B) Quantitative image analysis of AcGFP alone (n = 28), AcGFP-α-Syn-N-KQ (n = 27) and AcGFP-α-Syn-N (n = 30) accumulation at the plasma membrane. Protein localization was quantified by ImageJ software. Each dot shows the plasma membrane:cytosol intensity ratio. Bars, mean ± SEM. ***P < 0.001, Kruskal-Wallis followed by Dunn's multiple comparison tests.

We recently demonstrated that PA is intensely produced in Neuro-2a neuroblastoma cells during neuronal differentiation [24]. Moreover, PA generated by PLD is involved in neurite outgrowth [22]. Thus, we finally attempted to detect PA produced in Neuro-2a cells during serum starvation-induced neuronal differentiation. Interestingly, we found that AcGFP-α-Syn-N was located in the peripheral regions (close to the plasma membrane) of neurites growth cones (plasma membrane/cytosol intensity ratio: 1.65 ± 0.03 (mean ± SEM)) (Fig. 3A and B). However, AcGFP alone (plasma membrane/cytosol intensity ratio: 1.13 ± 0.05 (mean ± SEM)) and AcGFP-α-Syn-N-KQ (plasma membrane/cytosol intensity ratio: 1.02 ± 0.03 (mean ± SEM)) failed to exhibit such localization (Fig. 3A and B), suggesting that PA is produced in peripheral regions in the growth cone during neuronal differentiation. AcGFP-α-Syn-N partially co-localized with filamentous actin (F-actin) (Fig. 3A), which is regulated by PLD in the peripheral domains of the growth cone [22].

### 3.4 α-Syn-N detects PA generated by PLD in neuronal growth cones

**Fig. 4:**
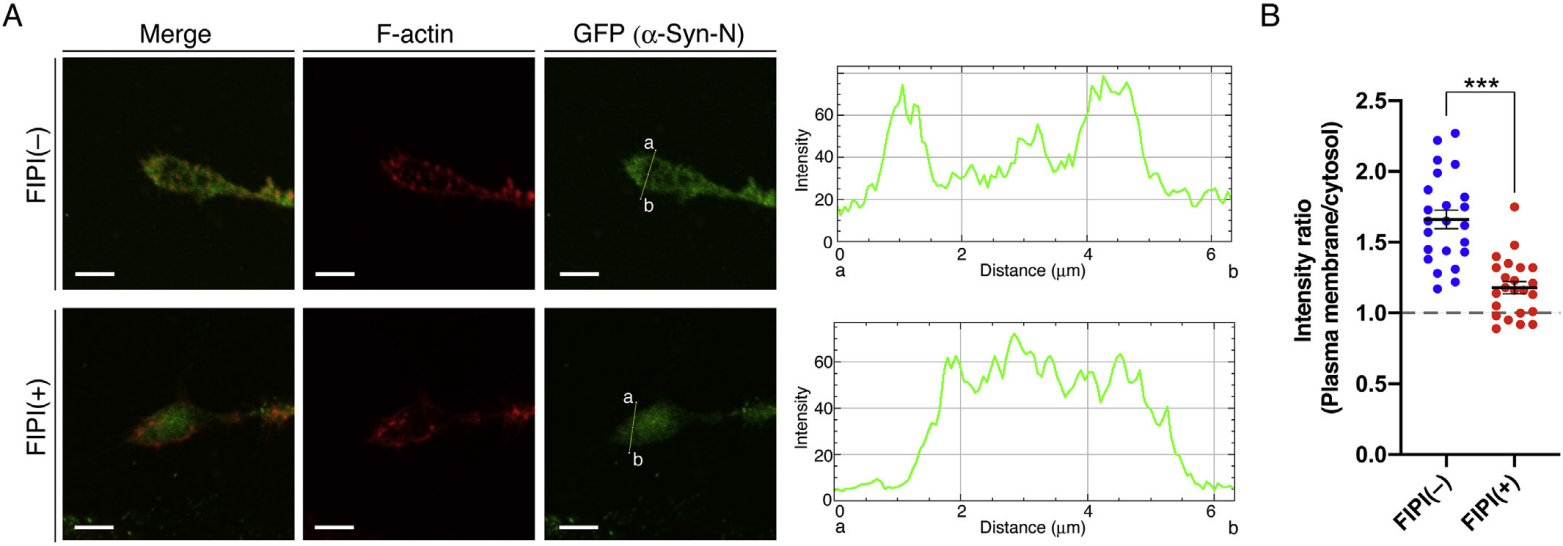
Effect of a PLD inhibitor on α-Syn-N localization at the growth cone. (A) Neuro-2a cells were cultured in serum-free DMEM for 48 h and then treated with either 750 nM FIPI or DMSO for 4 h. The localization of AcGFP-α-Syn-N in the growth cone of Neuro-2a cells was observed. Protein localization was quantified by ImageJ software. Representative data from three independent experiments are shown. Scale bars, 5 μm. (B) Quantitative image analysis of AcGFP-α-Syn-N accumulation at the plasma membrane (peripheral regions) in the absence (n = 23) and presence (n = 22) of FIPI. Each dot shows the plasma membrane:cytosol intensity ratio. Bars, mean ± SEM. ***P < 0.001, Kolmogorov–Smirnov test.

We next determined what enzyme(s) contributed to the PA production. Neuro-2a cells were treated with a PLD inhibitor (5-fluoro-2-indolyl deschlorohalopemide (FIPI)). Although AcGFP-α-Syn-N was strongly localized in the peripheral regions of growth cones in the absence of FIPI (plasma membrane/cytosol intensity ratio: 1.66 ± 0.06 (mean ± SEM)), the inhibitor (750 nM) almost completely inhibited the peripheral localization of AcGFP-α-Syn-N (plasma membrane/cytosol intensity ratio: 1.18 ± 0.04 (mean ± SEM)) (Fig. 4A and B), indicating that PLD primarily contributed to PA production in the region. Moreover, the results demonstrated that the peripheral localization of AcGFP-α-Syn-N occurs in a PA production-dependent manner, strongly suggesting that α-Syn-N recognized endogenously generated PA. Because FIPI nearly completely inhibited the peripheral localization of α-Syn-N, the contribution of DGK to PA generation in the region is likely small.

## 4 Discussion

In the present study, we first established an inactive PA sensor (α-Syn-N-KQ) as a negative control by changing all (eleven) lysine residues, which are predicted to be important for PA binding [28], to glutamine residues (Fig. 1A). α-Syn-N-KQ indeed lost PA-binding activity *in vitro* (Fig. 1B and C) and in cells (Fig. 1D and E). Because the predicted secondary structures of α-Syn-N and α-Syn-N-KQ were almost the same (Fig. 1A), α-Syn-N-KQ can be a useful negative control of α-Syn-N. We next determined that α-Syn-N, but not α-Syn-N-KQ, detected PA in macrophagic phagosomes (Fig. 2), in which PA is well known to be enriched [1,17]. Therefore, α-Syn-N is a reliable sensor for endogenous PA in cells.

Axon growth is driven by the forward movement of a growth cone, which consists of a central domain rich in microtubules and a peripheral domain enriched in actin filaments [29]. The distribution of PA in the neuronal growth cone remains unclear to date. In the present study, we found for the first time that α-Syn-N, but not α-Syn-N-KQ, detected PA in the F-actin-rich peripheral regions (close to the plasma membrane) in the neurite growth cones (Fig. 3).

FIPI almost completely inhibited the localization of α-Syn-N at the peripheral regions in neurite growth cones (Fig. 4), indicating that PLD mainly contributes to PA production at the growth cone. Remodeling the cytoskeleton is important for growth cone functions and has been suggested to be regulated by PA. For example, Ammar et al. demonstrated that PLD correlates with F-actin formation and neurite outgrowth [21]. Our findings further demonstrated the functional linkages among PA, PLD, cytoskeleton (F-actin) remodeling and neurite outgrowth/growth cone formation. Although 16:0/16:0-PA was highly generated in Neuro-2a neuroblastoma cells during neuronal differentiation, FIPI did not substantially reduce the PA amount [24]. Therefore, PLD probably generated a relatively small amount of PA in restricted regions (growth cones).

Even in the presence of FIPI, some α-Syn-N in the peripheral regions of the growth cone remained (plasma membrane/cytosol intensity ratio: 1.15 (not 1.00)) (Fig. 4). Thus, other PA-producing enzymes may produce PA in the peripheral regions. Previous studies reported that DGKζ regulated neurite outgrowth and co-localized with F-actin [30] and that the isozyme controlled the maintenance of the actin-rich spine [31]. We reported strong expression of DGKζ in Neuro-2a cells [24]. Moreover, overexpressed DGKζ was partly co-localized with α-Syn-N in the growth cone (Suppl Fig. 1). However, because long-term treatment with a DGKζ-specific siRNA also attenuated neurite formation [24] and a DGKζ-specific inhibitor is not available, it was difficult to analyze the contribution of DGKζ. In addition to DGKζ, DGKδ is abundant in the cells [24]. However, DGKδ did not produce considerable PA during the neuronal differentiation [24]. DGKβ also plays an important role in neurite outgrowth and spinogenesis through F-actin cytoskeleton remodeling [23,25]. However, because the expression level of DGKβ was low in Neuro-2a cells [24], DGKβ would not contribute substantially to PA generation in the cells. Therefore, among the DGK isozymes, DGKζ may generate PA at the growth cone during neuronal differentiation in Neuro-2a cells. However, further studies are needed to address this question.

α-Syn substantially bound to 18:1/18:1-PA, 16:0/18:1-PA and 16:0/16:0-PA [19]. PLD hydrolyses PC to produce PA [9], and PC contains a variety of molecular species including 18:1/18:1-, 16:0/18:1- and 16:0/16:0-PC [32]. Therefore, α-Syn-N probably detected 18:1/18:1-, 16:0/18:1- and 16:0/16:0-PA derived from PC in differentiated Neuro-2a cells. On the other hand, although α-Syn could not detect it in the present study, DGKζ generates 16:0/16:0-PA in Neuro-2a neuroblastoma cells during neuronal differentiation [24]. Unlike PLD, DGK isozymes produce distinct PA species [33]. For example, we reported that DGKδ produced 14:0/16:0-, 14:0/16:1-, 16:0/16:0-, 16:0/16:1-, 16:0/18:0- and 16:0/18:1-PA species in C2C12 myoblast cells [34] and 18:0/22:6-PA in the brain [Lu, Q. and Sakane, F., unpublished work]. In the DGKη-deficient mouse brain, 18:1/18:2-, 18:0/20:3-, 18:0/22:5-, 18:0/22:4- and 18:0/22:3-PA were significantly reduced [35]. Moreover, with respect to the targets of PA species, we recently found several PA species-selective targets in addition to α-Syn [19]. For example, the creatine kinase-muscle type selectively associated with 16:0/16:0-, 16:0/18:1-, 18:0/18:0- and 18:1/18:1-PA [36]. Moreover, Praja-1 E3 ubiquitin-protein ligase most strongly bound to 18:0/22:6-PA [Lu, Q. and Sakane, F., unpublished work]. l-lactate dehydrogenase A selectively interacted with 18:0/20:4- and 18:0/22:6-PA (Hoshino, F. and Sakane, F., unpublished work). Thus, a PA species sensor panel composed of many PA-binding proteins that selectively and characteristically recognize a variety of PA species is useful. Such a PA sensor panel could efficiently sense PA species produced in various cells in response to diverse cell stimuli. The panel may be able to distinguish PA produced by different PA-generating enzymes, for example, PLD-produced PA (mainly 16:0/18:1-PA species), DGKδ-produced PA (18:0/22:6-PA) and DGKζ-produced PA (primarily 16:0/16:0-PA species) in neurons.

In the present study, we provided a reliable PA sensor, α-Syn-N, which recognizes endogenous PA in cells. Moreover, we visualized PA in neurite growth cones for the first time using α-Syn-N and demonstrated that the PA was mainly generated by PLD. Our findings using the α-Syn-N sensor provide novel insights into PA distribution during neuronal differentiation. α-Syn-N is a useful sensor for endogenous PA, which plays important roles in a variety of physiological and pathological events.

## Declaration of competing interest

The authors declare no conflicts of interest associated with the contents of this article.
