# Supplementary material for: Cellular phosphatidic acid sensor, α-synuclein N-terminal domain, detects endogenous phosphatidic acid in macrophagic phagosomes and neuronal growth cones
Source: Biochem Biophys Rep. 2020 May 20;22:100769. doi: 10.1016/j.bbrep.2020.100769 (PMC7261706; doi:10.1016/j.bbrep.2020.100769)
Supplement: Multimedia component 1 [file mmc1.pdf]

Supplemental Materials

Cellular phosphatidic acid sensor, α-synuclein N-terminal domain, detects endogenous phosphatidic acid in macrophagic phagosomes and neuronal growth cones

Haruka Yamada, Fumi Hoshino, Qiang Lu and Fumio Sakane *

Department of Chemistry, Graduate School of Science, Chiba University, Chiba 263-8522, Japan

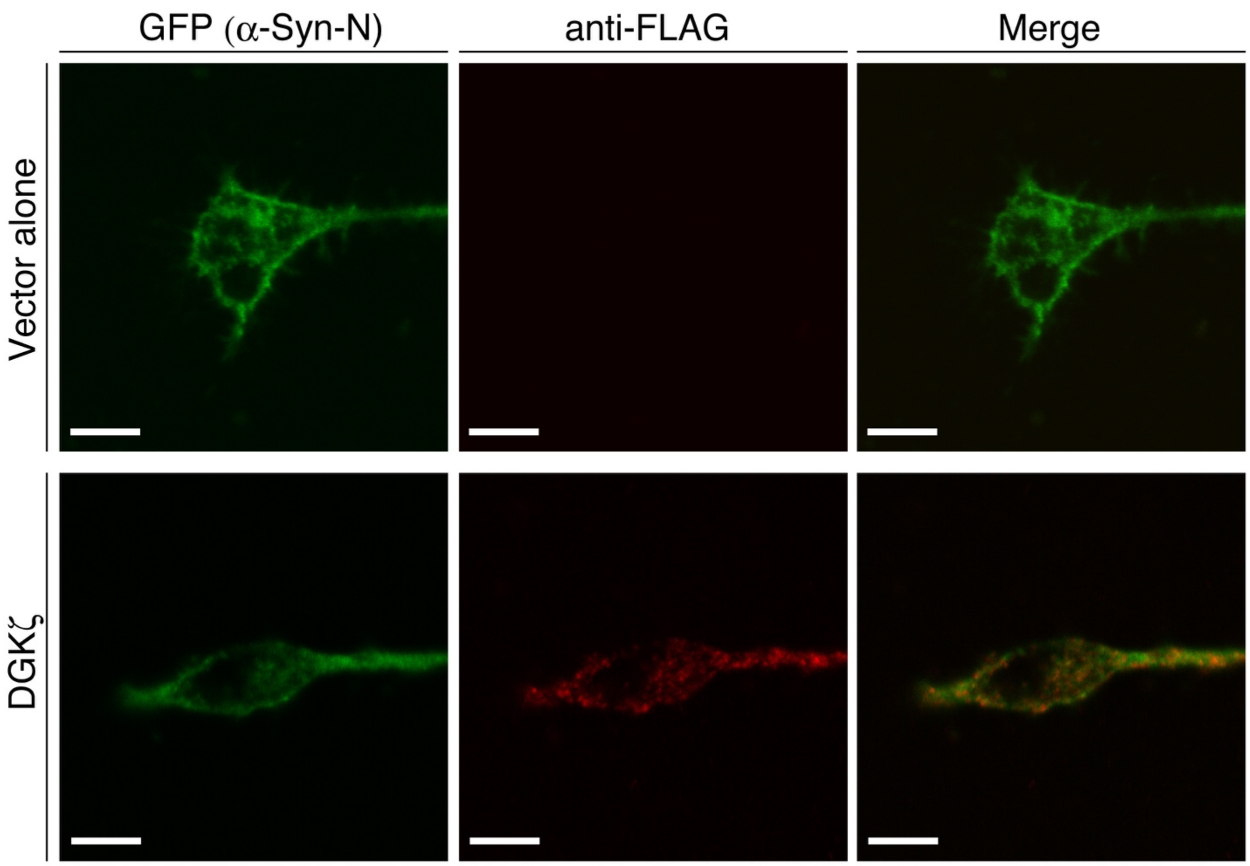

Suppl. Fig. 1. Localization of AcGFP-α-Syn-N and 3×FLAG-DGKζ in the growth cone of Neuro-2a cells

pAcGFP-α-Syn-N and either p3×FLAG vector alone or p3×FLAG-DGKζ were co-transfected into Neuro-2a cells. After 24h, the cells were cultured in serum-free DMEM for 48 h and then cells were stained with a mouse anti-FLAG monoclonal antibody and Alexa Fluor 594 goat anti-mouse IgG. Representative data from three independent experiments are shown. Scale bars, 5 μm.
